# Adaptation of ACMG-ClinGen Technical Standards for Copy Number Variant Interpretation Concordance

**DOI:** 10.3389/fgene.2022.829728

**Published:** 2022-03-10

**Authors:** Kuo Zhang, Guigao Lin, Dongsheng Han, Yanxi Han, Rongxue Peng, Jinming Li

**Affiliations:** National Center for Clinical Laboratories, Institute of Geriatric Medicine, Chinese Academy of Medical Sciences, Beijing Hospital/National Center of Gerontology, Beijing, China

**Keywords:** copy number variant, classification and interpretation, concordance, ACMG-ClinGen technical standards, inter-laboratory

## Abstract

This study aimed to evaluate inter-laboratory classification concordance for copy number variants (CNVs) with a semiquantitative point-based scoring metric recommended by the American College of Medical Genetics and Genomics (ACMG) and Clinical Genome Resources (ClinGen). A total of 234 CNVs distributed by the National Center of Clinical Laboratories (NCCLs), and 72 CNVs submitted by different laboratories, were distributed to nine clinical laboratories performing routine clinical CNV testing in China and independently classified across laboratories. The overall inter-laboratory complete classification concordance rate of the 234 distributed CNVs increased from 18% (41/234) to 76% (177/234) using the scoring metric compared to the laboratory's previous method. The overall inter-laboratory complete classification concordance rate of the 72 submitted CNVs was 65% (47/72) using the scoring metrics. The 82 variants that initially did not reach complete concordance classification and 1 additional CNV deletion were reviewed; 34 reached complete agreement, and the overall post-review complete concordance rate was 85% (260/306). Additionally, the overall percentage of classification discordance possibly impacting medical management [i.e., pathogenic (P) or likely pathogenic (LP) vs. variant of uncertain significance (VUS)] was 11% (35/306). The causes of initial and final discordance in the classification were identified. The ACMG-ClinGen framework has promoted consistency in interpreting the clinical significance of CNVs. Continuous training among laboratories, further criteria and additional clarification of the standards, sharing classifications and supporting evidence through public database, and ongoing work for dosage sensitive genes/regions curation will be beneficial for harmonization of CNVs classification.

## Introduction

Copy number variants (CNVs) are a class of human genetic variations comprising gains (duplications and triplications), losses (deletions), or complex rearrangements. CNVs can disrupt the coding region or alter gene dosage and contribute to a broad range of genetic disorders, including intellectual disability (ID) and other neurodevelopmental disorders (NDDs), as well as multiple congenital anomalies (Nowakowska et al., 2017; Riggs et al., 2020a). In patients with several other diseases, such as deafness, renal disorder, blindness, and complex phenotype, CNVs reportedly play an important role (Pfundt et al., 2017). In contrast, there are a proportion of benign CNVs in the human genome which are not enriched in individuals with abnormal phenotype and are repeatedly found in normal control populations (Tsuchiya et al., 2009; Nowakowska et al., 2017). Understanding the potential clinical significance of CNVs is crucial for their medical management. However, the frequency data of CNVs from healthy individuals remains limited, and novel CNVs are continually discovered; accurate and consistent determination of the clinical significance of individual rare CNVs across laboratories is challenging (Kearney et al., 2011).

In recent years, next-generation sequencing (NGS)-based analyses have been increasingly utilized in inherited disease diagnosis, enabling the simultaneous detection of more sequence variants and CNVs in a single test, which has further increased the possibility that clinical laboratories interpret the same variants differently (Hehir-Kwa et al., 2015). To provide guidance to clinical laboratories towards the conduction of more consistent variant classifications, in 2015, the American College of Medical Genetics and Genomics (ACMG) and the Association for Molecular Pathology (AMP) published the variant classification guidelines (Richards et al., 2015), providing a framework for sequence variants clinical interpretation. The guidelines and subsequent guidances in both general and specific disease/gene–disease contexts (www.clinicalgenome.org, Accessed in 2021, oct) have helped to significantly reduce discrepancies in the clinical interpretation of sequence variants (Richards et al., 2015; Amendola et al., 2016; Garber et al., 2016; Niehaus et al., 2019; Amendola et al., 2020). Nonetheless, the implementation of these guidelines has not eliminated issues, and the laboratories may continue to disagree regarding the clinical interpretation of sequence variants (Amendola et al., 2020; Wain et al., 2020). Similarly, efforts have also been engaged towards the establishment of a more consistent interpretation of CNVs. The previous ACMG technical standard (Kearney et al., 2011) was published in 2011 to provide assistance to laboratories in the evaluation of CNVs but did not address the methods that can be adopted to categorize and weigh each type of evidence; therefore, clinical laboratories had to develop their own methods for CNVs classification. Thereafter, in 2020, based on the previously established standard, a semiquantitative point-based scoring metric was presented in ACMG and the Clinical Genome Resources (ClinGen) (Riggs et al., 2020a) technical standards, intending to further refine the process for CNVs classification and to promote consistency in interpretation and reporting of CNVs. The relative weight was assigned to each evidence category, consisting of genomic content (Section 1); evaluation of dosage sensitivity (Section 2); gene number (Section 3); evidence based on cases in the published literature, public databases and/or internal lab data, and case–control and population evidence (Section 4); and inheritance pattern and family history for the patient under investigation (Section 5). The point values were summed to deduce one of the following five classifications: pathogenic (P), likely pathogenic (LP), variant of uncertain significance (VUS), likely benign (LB), or benign (B); the classifications complied with the recommendations in sequence variant interpretation. Based on the scoring metrics, evaluation of a total of 111 CNVs by two independent reviewers was conducted, and 64.9% of the CNVs reached the same classifications (Riggs et al., 2020a). However, there are more classification-associated challenges for CNVs, which include limitations (e.g., data content, database structure, and accuracy interpreting the data) of the special database resources for CNV interpretation (e.g., DECIPHER and DGV) compared with sequence variant databases, limited evidence available for few CNVs, difficulty in performing comparison of the proband's CNV with previously published CNVs due to breakpoint uncertainty based on the application of different technologies, complexity of gain interpretation for unknown location and orientation, and so on (Brandt et al., 2020). Additionally, the ACMG-ClinGen technical standards remain insufficient in terms of the education, familiarity, and experience among researchers regarding the use of scoring metrics. The extent to which classification differences exist and the factors contributing to discordance remain unknown.

The present study carefully compared the inter-laboratory classification concordance assigned to CNVs across nine clinical laboratories with the ACMG-ClinGen standards. The aim of the study was to evaluate the consistency of the use of the ACMG scoring metrics and the subsequent pathogenicity classification and to explore the variance in the use and interpretation of points-based scoring metrics. The factors contributing to the classification discordance were further investigated. The results may help clinical laboratories in the development and establishment of more consistent, transparent evidence evaluation practices using the CNVs scoring metrics.

## Materials and Methods

### Selection of the Participating Laboratories

The National Center of Clinical Laboratories (NCCLs) organized nine Chinese clinical laboratories (seven associated with hospitals and two associated with commercial entities) to evaluate the concordance of CNVs interpretation using the ACMG-ClinGen scoring metrics and subsequent pathogenicity classification. These nine laboratories perform CNVs analysis using chromosomal microarray (CMA) or genome sequencing for routine clinical testing. Each laboratory presented with at least 5 years of experience in CNVs analysis conducted for the detection of disease-causing losses and gains across the genome. Five laboratories each presented with analysis of samples from more than 10,000 postnatal or prenatal individuals, and four laboratories each presented with analysis of 6,000–10,000 postnatal or prenatal samples. These laboratories have established their own standard operating procedures (SOP) of interpreting and reporting CNVs according to the ACMG-ClinGen technical standards, and they reported the assessment of the pathogenicity of CNVs following these standards.

### CNV Set Creation, Distribution, and Classification

In 2019, nine clinical laboratories were first advised to evaluate CNVs, as per their typical procedures (mainly according to CNV size, internal and external databases, genomic content, medical literature, and so on) before issuance of the ACMG-ClinGen technical standards. Seventy-seven nonrecurrent CNV deletions, 63 nonrecurrent CNV duplications, and 47 recurrent CNVs (deletions and duplications for the same CNV) for a total of 234 CNVs were distributed to all clinical laboratories by email. The CNVs were selected from ClinGen, DECIPHER, published literature, or CNVs NCCLs previously testing. They spread over all human autosomes and sex chromosomes, covering each evidence category from Section 1 to Section 4 as possible. Size distributions were as follows: 8 CNVs are greater than 10 Mb, 22 CNVs are 5–10 Mb, 127 CNVs are 1–5 Mb, and 77 CNVs are smaller than 1 Mb. In 2020, after the ACMG-ClinGen standards were published, the CNVs for which one laboratory showed disagreement of classifications with those reported by others (93 deletions and 100 duplications in total) were selected for redistribution to the same nine laboratories, and the laboratories were asked to evaluate them using the ACMG-ClinGen scoring metrics. They were also asked to document the scoring metrics sections that were adopted for classification and weights for each category of evidence.

To further understand what are the problems in using scoring metrics in the laboratories' routine testing, seven laboratories provided a range of CNVs (36 duplications and 36 deletions) for classification with varying degrees of difficulty they thought. Size distributions of 72 CNVs were as follows: 7 of them are greater than 10 Mb, 13 of them are 5–10 Mb, 28 of them are 1–5 Mb, and 24 of them are smaller than 1 Mb. The chromosomal location of these submitted CNVs were not the same as the 234 CNVs that were distributed by NCCLs. The laboratories submitting CNVs were also asked to provide the reasons of the interpreting difficulty they though and any internal evidence that they considered to interpret the CNVs, for example, phenotype, parental inheritance, and testing method, if any. Along with the 193 discordant classification CNVs in 2020, such CNVs with any internal evidence were shared with other laboratories without the submission of laboratory classification. These shared CNVs were only evaluated using the ACMG-ClinGen scoring metrics by all other laboratories. Independent classifications with evidence codes and weights were obtained for each CNV to further investigate the factors contributing to the classification discordance.

### Analysis of Inter-Laboratory Concordance and Further Classification Concordance

The present study compared the 234 distributed CNV classifications which were submitted by the laboratories; furthermore, it evaluated the inter-laboratory classification concordance difference when using the laboratory’s previously established method compared with when using the ACMG-ClinGen CNV scoring metrics. All nine laboratories used the same classification that was considered the complete five-category concordance across laboratories. Considering that the variants classified as P *versus* LP or B *versus* LB would be managed using the same clinical recommendations, the classification difference across laboratories was defined as the “confidence differences” category. These two categories were considered as clinically meaningful concordance. The overall inter-laboratory complete five-category concordance rate and clinically meaningful concordance rates were assessed. The study further examined differences in the rate of conflicts that might impact medical management (i.e., P or LP vs*.* VUS or LB or B across laboratories) or conflicts that might impact the return of results (ROR, i.e., VUS vs*.* LB or B across different laboratories). Then, it further explored the rate difference observed in different CNV types (deletions and duplications) and different subsets (recurrent and nonrecurrent). The overall classification concordance among the 72 submitted CNVs was also assessed. A chi-square test with a statistically significant threshold of *p* < .05 was used to evaluate the rate differences.

The classifications were verified to identify the source of the discrepancies. The possible problems and errors in the process of using the scoring metric system have been summarized. Then the relevant information was shared with the laboratories for further classification concordance. The CNVs that initially did not reach complete five-category concordance classification across nine laboratories using the ACMG-ClinGen CNV scoring metrics (i.e., discrepant classifications) were selected and provided to all laboratories, and information on the cases in the published literature or public databases considered by laboratories for each CNV were also provided to laboratories for further classification concordance. Each laboratory was finally asked to re-evaluate the categories of evidence, to correct errors, and to provide a final classification. Inter-laboratory classification concordance was then evaluated.

## Results

### Inter-Laboratory Classification Concordance Using the ACMG-ClinGen CNV Scoring Metrics System

**FIGURE 1:**
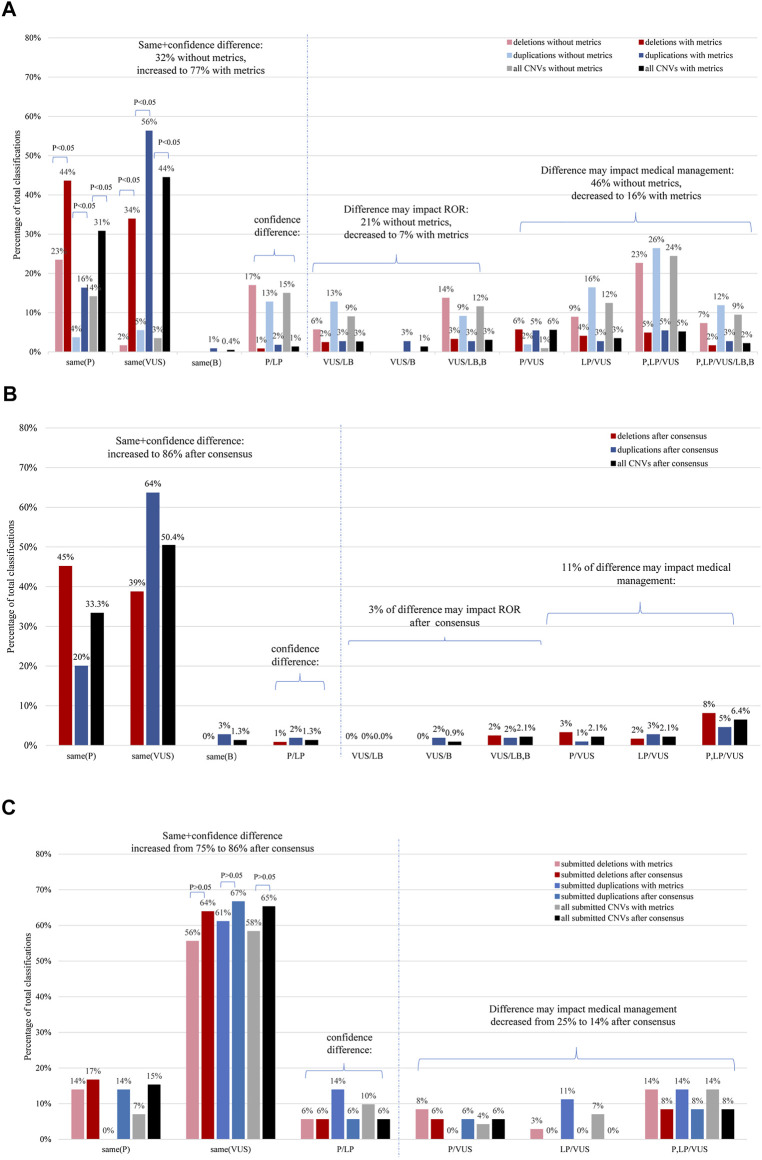
Distribution of CNV classification comparisons according to the extent of differences using the CNV scoring metrics. **(A)** Inter-laboratory concordance of the distributed CNVs. The graph illustrates a comparison of the percentage of classifications of the same CNVs, based on either the original data or those obtained using the CNV scoring metrics (N = 234). **(B)** Inter-laboratory classification concordance of deletions and duplications which underwent further evaluation for the distributed CNVs (N = 234). **(C)** The inter-laboratory classification concordance of deletions and duplications was compared before and after conduction of further evaluation for the submitted CNVs (N = 72). The results are shown for deletions (red), duplications (blue), and for all CNVs combined (gray or black). ROR, return of result; P, pathogenic; LP, likely pathogenic; VUS, uncertain significance; LB, likely benign; B, benign. Confidence difference implies that the classification difference does not affect medical management. *p < .05 means there was a statistical difference.

For the 234 distributed CNVs, the inter-laboratory classification concordance before and after using the ACMG-ClinGen CNV scoring metrics is summarized in Figure 1A. The overall complete five-category concordance rate across nine laboratories involved in the classification of each CNV was 18% (41/234), according to the laboratory’s previously established method. After the 193 CNVs with discrepant classifications based on the original clinical laboratory classification were re-evaluated using the ACMG-ClinGen scoring metrics by all nine laboratories, an additional 136 variants showed agreement of classifications, and the complete five-category concordance rate increased to 76% (177/234) (chi-square test; *p* < .05); the VUS-category concordance rate was the highest (44%, 104/234). During grouping of the confidence difference category (P vs. LP or B vs. LB), the clinically meaningful concordance rate increased from 32% (76/234) to 77% (180/234) (chi-square test; *p* < .05). The classification differences more likely to impact medical management (P or LP *vs.* any of VUS, LB, and B) decreased from 46% (107/234) to 16% (38/234) (chi-square test; *p* < .05).

The outcomes of recurrent and non-recurrent subsets classification have been presented in Supplementary Figures S1A, B respectively. With application of the ACMG/ClinGen scoring metrics, the rate of the complete five-category concordance showed differences in the CNV subsets, going from high to low: recurrent deletions (91%, 43/47), nonrecurrent duplications (75%, 47/63), recurrent duplications (72%, 34/47), and nonrecurrent deletions (69%, 53/77). There was no statistically significant difference between the overall deletion and duplication classification (77 vs. 74%; chi-square test, *p* > .05). The rate of complete VUS-category concordance of non-recurrent CNVs (60%, 84/140) was markedly higher than that of recurrent CNVs (21%, 20/94) (chi-square test; *p* < .05). Recurrent deletions and recurrent duplications were more likely to be classified as complete P (59%, 56/94) than non-recurrent (11%, 16/140) (chi-square test; *p* < .05).

For a subset of submitted CNVs with varying degrees of difficulty, the overall rate of complete five-category concordance was 65% (47/72) [no significantly difference from that of the 234 distributed CNVs (76%,177/234); chi-square test, *p* > .05] based on application of the ACMG-ClinGen scoring metrics (Figure 1C). There was no statistically significant difference in the clinically meaningful concordance rates between the classifications of deletions and duplications (70%, 25/36 vs. 61%, 22/36; chi-square test, *p* > .05). Twenty-five percent (18/72) of CNV classifications showed a discordance that would impact medical management (nine deletions and nine duplications).

### Reasons for Discordance and Problems Regarding Laboratory Usage Across the Scoring Metrics

A total of 82 discrepant classifications were achieved using the ACMG-ClinGen CNV scoring metrics: 56 showed disagreement of classifications which could impact medical management, 16 showed disagreement of classification which could impact ROR, and 10 showed discordance between P and LP. The reasons and problems for discordance were identified during the analysis of evidence and weights submitted by laboratories for the 82 discordant CNVs, which have been presented in Table 1.

**TABLE 1:** Reasons and problems for classification discordance.

Evidence category	Reasons and problems
Section 1	(1) Misapplication of 1A for CNVs completely void of gene content, or misapplication of 1B for CNVs containing protein-coding genes or other known functionally important elements
Section 2	(1) Error evaluation of the established dosage-sensitive genes/genomic regions, or evaluation of benign genomic regions
	(2) The evaluated region with the AR gene, for which loss of function is an established disease mechanism, was erroneously used for category 2A directly, and no further evaluation for the whole region was conducted
	(3) Data on the reported syndrome region in Online Mendelian Inheritance in Man (OMIM) or other sources used without curation by ClinGen was erroneously used for category 2A
	(4) Disagreement over the usage of a few categories or the assignment of different points for certain categories, for example, 2C-1
	(5) Disagreement on how the patient's phenotype was interpreted, for example, the use of 2K (0.45 points) or 2J (0 point) when a copy number gain breakpoint was observed for the established HI genes
Section 3	(1) Variations in the evaluation of a considerable gene family and doubts arising in the consideration of one gene or multiple genes
Section 4	(1) Variations in selecting a gene of interest within the CNV if there is no compelling region level evidence available, or if the region level evidence alone is insufficient
	(2) Differences in the ability to scrutinize/examine case-level data in other data sources, such as published literature, public databases (e.g., DICIPHER, Clinvar, and so on), and/or internal laboratory data, and variations in the usage of other reported probands
	(3) Variations in using specific case-level data in other data sources as evidence
	(4) Variations in weight upgradation and downgradation for certain categories
	(5) Different applications of case–control and population evidence
	(6) Variations in determining a specific phenotype category for usage (highly specific phenotype or case with high genetic heterogeneity) and misapplication of certain categories; for example, cases with unknown inheritance were used in the establishment of nonspecific phenotypes (category 4E)
	(7) Problems associated with the selection of individuals for obtainment of evidence towards observed segregation
	(8) Variations in evaluating confirmed or assumed *de novo* variations in considering the specificity of phenotype led to differences in the weight assigned
	(9) Variations in evaluating inheritance in family members when the CNV region is imprinted or exhibits reduced penetrance

For 13 CNVs, the classification discordance was attributable to the error in usage of certain categories in some laboratories, for example, misapplication of category 1B for CNV in the imprinted region (2 of 13 CNVs), error evaluation of the established dosage-sensitive genes/genomic regions or benign genomic regions (8 of 13 CNVs), direct error usage of category 2A for CNV with the LOF AR gene without further evaluation for the whole region and genes in the region (2 of 13 CNVs), or direct error usage of the reported syndrome region in the literature without curation (1 of 13 CNVs).

Different points assigned for a few categories were also attributable for discordance; for example, different points assigned for 2C-1 were involved in two CNVs. When a copy number gain breakpoint was observed for the established HI genes, there was disagreement on the use of 2K (0.45 points) or 2J (0 point) in how the patient's phenotype was interpreted. For four CNVs, the classification discordance was attributable to variations in counting a large gene family as one gene or multiple genes (Section 3—evaluation of gene number). For the majority of discordant CNVs (76%, 62/82), the most common reason for discordance was the different application of categories outlined in Section 4 (detailed evaluation of genomic content using cases). For six CNVs, different applications of case–control and population evidence (4L-4O) contributed to classification discordance. For example, variation in weight was assigned if a variant was observed in the general population but at a frequency lower than 1%. One laboratory always assigned less weight according to the number of people in the DGV-gold and gnomAD datasets. For other CNVs, the existence of more than one reason and multiple problems resulted in different applications of evidence categories in Section 4, including differences in the ability to scrutinize case-level data in other data sources, variations in utilization of appropriate case-level data, variations in considering the specificity of phenotype, and so on.

### Final Classifications and Outcomes for Discordant CNVs

The reasons and problems associated with classification discordance were explained and returned to the participating laboratories. Subsequently, 82 CNVs with discordant classifications were re-evaluated by the participating laboratories. One deletion, *17p13.3 (1551800–2264023)×1* (GRCh37), was also re-evaluated despite its complete VUS category concordance across laboratories because we found it can be classified as pathogenic CNV according to weight assigned by us. The approach to the variant classification review is presented in Supplementary Figure S2.

The inter-laboratory classification concordance of deletions and duplications after re-evaluation for the distributed CNVs is summarized in Figure 1B and Supplementary Figures S1C,D. The overall complete concordance across laboratories involved in the classification of each CNV increased to 85% (199/234) after a consensus review. When grouping the confidence difference (P *vs.* LP), the clinically meaningful concordance rate increased to 86% (202/234).

The inter-laboratory classification concordance of deletions and duplications before and after re-evaluation of 72 submitted CNVs are summarized in Figure 1C. Overall complete concordance across laboratories involved in the classification of each submitted CNV increased to 85% (61/72) after a consensus review. When grouping the confidence difference (P vs. LP), the clinically meaningful concordance rate increased to 86% (62/72).

**FIGURE 2:**
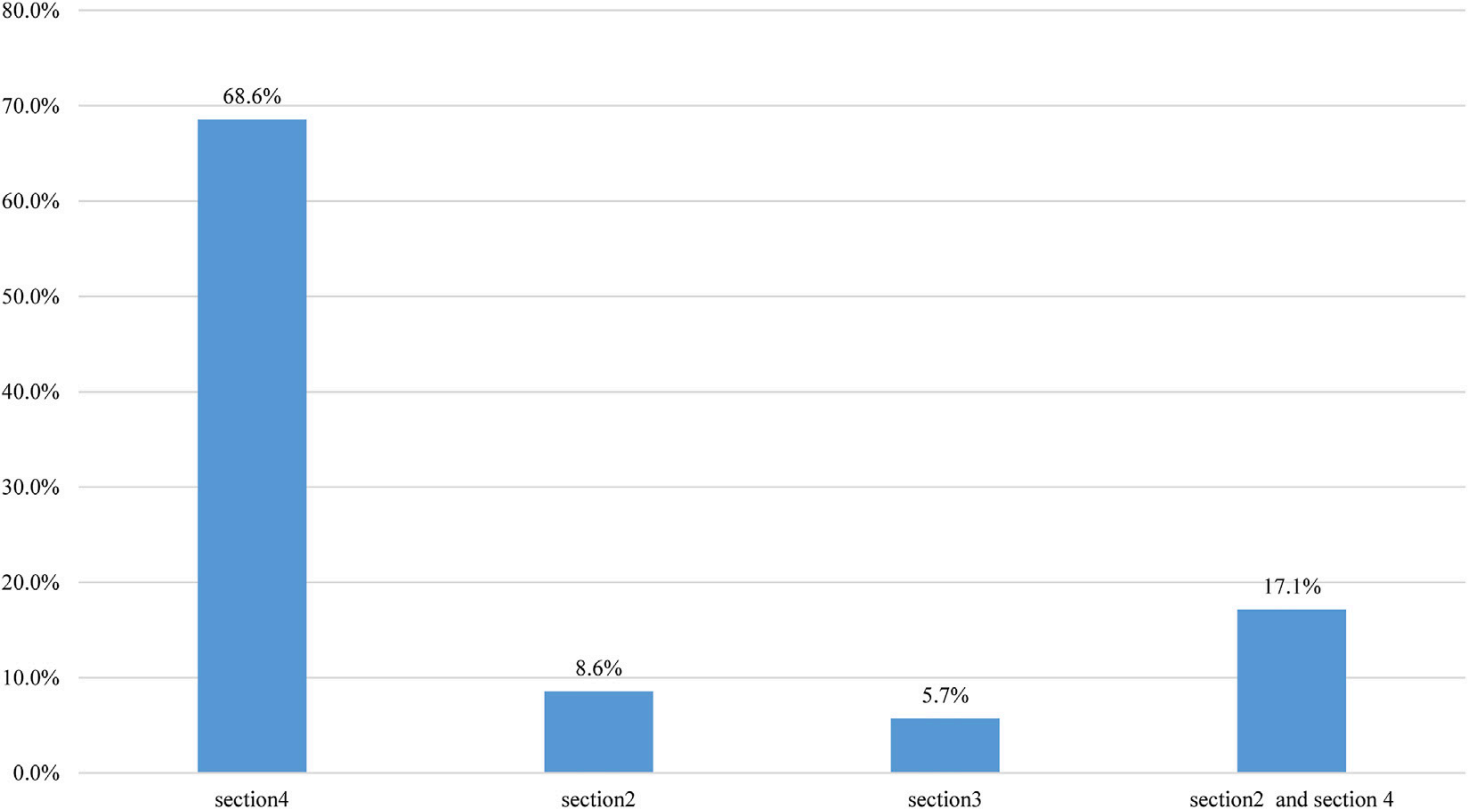
The reasons for classification discordance after consensus reason. The reasons were summarized according to the categories of evidence submitted by laboratories for the 35 CNVs, with classification discordance possibly impacting medical management. The column “section 2 + section 4” indicates that the discordance is attributable to usage differences in both sections 4 and 2.

The final classifications of the 82 discordant CNVs are summarized in Table 2. A total of 306 CNVs with final concordant and discordant classifications are shown in Supplementary Tables S1–S5. Of the 83 re-evaluated CNVs, a difference of 42% (35/83) may impact medical management (i.e., P or LP vs. VUS) after a consensus review. The overall classification discordance rate possibly impacting medical management was 11% (35/306). Notably, *17p13.3* (*1551800–2264023×1* (GRCh37) was classified in a discordant manner after re-evaluation (P vs. VUS). The reasons for the classification discordance of 35 CNVs after consensus have been summarized in Figure 2. The discordance was mainly attributable to case differences and/or scoring differences in Section 4 alone (68.6%, 24/35), followed by usage differences in both Section 2(evaluation of dosage sensitivity) and Section 4 (17.1%, 6/35).

**TABLE 2:** Final classifications of discordant CNVs.

Initial discordant classification	Number of CNVs	Final complete five-category concordance classification	Final discordant classification
Deletion CNVs			
P or LP	3		P or LP 3
P or VUS	10	VUS 6	P or VUS 3; P or LP *vs*. VUS 1
LP *vs*. VUS	6	P 1	P or LP *vs*. VUS 3; LP or VUS 2
P or LP *vs*. VUS	11	P 2	P or LP *vs*. VUS 7; P *vs*. VUS 2
Por LP *vs*. VUS *vs*. LB or B	2		P or LP *vs*. VUS 2
LB or VUS	3	VUS 3	
B or VUS	0		
LB or B *vs*. VUS	4	VUS 1	LB or B *vs*. VUS 3
Total number	39	13	26
Duplication CNVs			
P or LP	7	P 4	P or LP 3
P or VUS	6	P 3, VUS 1	P or LP *vs*. VUS 1; P or VUS 1
LP or VUS	7	VUS 3, P 1	P or LP *vs*. VUS 1; P or VUS 1; P or LP 1
P or LP *vs*. VUS	11	P 1, VUS 2	P or LP *vs*. VUS 5; P *vs*. VUS 1; LP *vs*. VUS 2
Por LP *vs*. VUS *vs*. LB or B	3	VUS 1	P or LP *vs*. VUS 1; LP *vs*. VUS 1
LB *vs*. VUS	3	VUS 2	LB *vs*. VUS 1
B *vs*. VUS	3	B 2	B *vs*. VUS 1
LB or B *vs*. VUS	3	VUS 1	LB or B *vs*. VUS 1; VUS *vs*. B 1
Total number	43	21	22

## Discussion

The establishment of the ACMG-ClinGen semi-quantitative point-based scoring metrics is an important step in improving CNVs classification consistency. The study evaluated the implementation of the ACMG-ClinGen standards in CNVs assessment and provided a concordance estimate for CNVs classifications across laboratories at the first instance of use.

As described in the *Results*, the increase of overall inter-laboratory complete classification concordance rate and the decrease the of classification differences affecting medical management with scoring metrics compared to without scoring metrics underscored the importance of a standardized, evidence-based approach for consistent CNV assessment across laboratories. In other words, implementation of point-based scoring metrics has provided guidance to laboratories towards the establishment of more consistent CNV classifications. Notably, the overall inter-laboratory complete classification concordance rate was relatively low (18%, 41/234) by using laboratory’s previous method compared to by using metrics (76%, 177/234) (Figure 1A). The main underlying reason may be attributable to the variations of previous methods in Chinese laboratories in considerations of CNV size, well-established syndromes, genomic content in CNV internal, comparison rule of CNV with external databases, gene inclusion principle, and so on. This effect is particularly pronounced for non-recurrent CNVs classification concordance (Supplementary Figures S1A,B). The original laboratory non-consistent classification (46%) was higher than the previously reported value (22.4%) (Riggs et al., 2020a). The main underlying reason may be attributable to a greater number of participating laboratories and a greater number of independent classifications in the present study than that previously reported. When using the ACMG-ClinGen scoring metrics, the initial overall complete concordance across laboratories for all CNVs was 73% (224/306), which was higher than the value (64.9%) previously reported (Riggs et al., 2020). This may be attributable to the fact that the participating laboratories have implemented scoring metrics for a certain period (at least half a year), familiarity and systems are constantly evolving, and such aspects are imperative for classification concordance. After a consensus review, discordance classification that was likely to impact medical management was mainly attributable to the variations in the ability to examine case-level data in other data sources (e.g., evaluation of significance, effect size, and clinical information) and variations in which case-level data could be used as evidence (e.g., overlapping degree in genomic content between the observed CNV with reported CNV). These underscore the need for ongoing education and familiarity with the ACMG-ClinGen technical standards, ongoing training in the use of genetic resources, usage of scoring categories, and evaluation of case-level data inter-/intra-laboratory. Another reason is the variation in weight assigned for certain categories, for example, category 4O when evaluating CNV overlapping with common population variation but at a frequency lower than 1%. Therefore, establishment of further criteria and additional clarification of the standards may help increase the inter-laboratory concordance rate.

Returning the reasons and problems for discordance to the participating laboratories and sharing of the evidence collected by laboratories for each discordant CNVs led to decrease in discordance of the analyzed CNVs, which underscored the importance of sharing CNV interpretation data to resolve discordance. Previous studies have demonstrated the value of sharing data for classifications and supporting evidence on sequence variant classification concordance (Rivera-Muñoz et al., 2018; Amendola et al., 2020). Ongoing and robust efforts to establish a classification consensus for sequence variants are being undertaken through consideration of the ClinGen programs (Harrison et al., 2018), and efforts to resolve variant classification discordances in the ClinVar database are also being performed (Rivera-Muñoz et al., 2018). Such efforts and ongoing work for sharing of data across laboratories should also be performed to harmonize CNVs classification. Additionally, interpretation of the pathogenicity of CNVs often relies on frequency information obtained from a healthy cohort and databases with previously reported CNVs. However, information on CNVs in public databases, such as DECIPHER and ClinVar, is relatively limited in interpretation of clinical significance and paucity of detailed evidence to support classifications. Laboratories should be encouraged to deposit their initial classification with supporting evidence as well as any relevant unpublished data into public resources, which may be used partly to inform clinical laboratory CNVs interpretations.

The results demonstrated that participating laboratories were most likely to agree on the classifications as VUS for non-recurrent CNVs compared with recurrent CNVs (Supplementary Figures S1A,B), which is not a remarkable observation, because the relative paucity of studies and cases of noncurrent CNVs are available in the published literature, public databases, or internal data, supporting them either benign or disease-causing classification. This is challenging for rare and non-recurrent variants because of their extensive spectrum of effects. Notably, complete VUS-category concordance across laboratories did not necessarily reflect the same final point values assigned to each CNV, especially for each piece of evidence in Section 4. We also found that a pathogenic CNV deletion (*17p13.3 (1551800–2264023) ×1* (GRCh37)) was classified as complete VUS-category concordance among laboratories in initial classification using scoring metrics, which was attributable to the fact that the gene of interest was not the *PRPF8* gene associated with dominantly inherited retinitis pigmentosa within the CNV, or inter-laboratory differences in the ability to examine case-level data in public sources. The lowest complete concordance of the benign group is not remarkable because the CNVs containing protein-coding genes could be classified as VUS category; however, it is not known whether the genes are dosage-sensitive, and much remains unknown about their function (Harrison et al., 2018). The ClinGen consortium is curating genes and regions of the genome by assessing whether evidence exists to support that genes/regions are dosage-sensitive (https://dosage.clinicalgenome.org/, Accessed in 2021, oct) (Riggs et al., 2012). However, the completely curated genes and genomic regions are limited; for the majority, there exists insufficient evidence to support a haploinsufficiency rating, and their role in a particular phenotype remains elusive. On the other hand, new and emerging evidence is not incorporated in a timely manner, and certain genes/regions are not re-evaluated on a periodic basis. These underscore the importance of ongoing work aimed at elucidating dosage-sensitive genes/regions curation, not only for potentially resolving discordance but also for understanding the roles of genes/regions in human health maintenance and disease development. As experience and knowledge accumulate gradually, CNVs initially classified as a VUS may be reclassified as either a benign or pathogenic category. Thus, the relevant laboratories should also strive towards the development of processes to document, to track, and to re-evaluate previously classified CNVs.

In the present study, a copy number loss *15q11.2 (22754322–23109890)×1* (GRCh37) (Supplementary Table S1) containing the *15q11.2* recurrent region (BP1-BP2) associated with the occurrence of neurodevelopmental disorders has been classified by various laboratories as a variant of VUS, LP, or P (6/1/2 laboratories, respectively), demonstrating that laboratories encountered confusion regarding the categorization of CNVs with reduced penetrance which are not rare in the population. A previous study (Jønch et al., 2019) has recommended that *15q11.2* deletion should be classified as “pathogenic of mild effect size” because it explains only a small proportion of the phenotypic variance in carriers and it is not worth discussion in the developmental clinic or the prenatal setting. Maya et al. (2020) proposed the introduction of a new, separate “high-frequency low-penetrant variants” category for variants with a penetrance below 10% and a frequency of over 0.1% in the healthy population. The ClinGen Low Penetrance/Risk Allele Working Group proposed the use of the descriptor “low penetrance” or “reduced penetrance” in addition to the primary variant classification term (e.g., pathogenic), irrespective of the availability of sufficient quantitative penetrance estimates (Riggs, et al., 2020b). The working group is also exploring whether the current standards/guidelines (Richards et al., 2015; Riggs et al., 2020a) used for variant interpretation can be adapted for the evaluation of low-penetrance variants. Laboratories should be aware of current and future proposals in terms of the classification and assessment of low-penetrance variants, and caution should be exercised before interpreting such CNVs. On the other hand, the establishment of a local CNV database for case–control populations would enable a better understanding of the penetrance of recurrent CNVs in specific populations (Maya et al., 2020).

The present study had its limitation. The variant set has a limited number of LB or B CNVs and does not represent all evidence categories, especially for deletions and duplications within the individual genes (category 2E in the deletion metric and category 2I in the duplication metric). However, this study systematically evaluates the implementation of the ACMG-ClinGen semiquantitative point-based scoring metric in the medical practice of CNVs assessment, provides a classification variability estimate across laboratories, and underscores reasons and problems likely to contribute to classification discordance.

The study also discovered that the interpretation of the same CNV by multiple clinical laboratories may differ, even when using the ACMG-ClinGen standards; this indicates that at least one interpretation must be wrong and may therefore lead to inappropriate medical intervention. Therefore, interpretation of the pathogenicity of CNVs remains challenging; additional studies and ongoing efforts are necessary to further improve CNV classification. The ACMG-ClinGen technical standards proposed the establishment of a quantitative scoring framework; variant classifications should be based on evidence; however, they must not be used as the sole evidence of abnormal phenotype; ascertainment of a given CNV of clinical consequence may depend on other factors, and laboratories should therefore establish specific reporting practices based on CNV classification and clinical context and indication for testing.

## Data Availability

The original contributions presented in the study are included in the article/Supplementary Material, further inquiries can be directed to the corresponding author.
