# Supplementary figures and images for: Adaptation of ACMG-ClinGen Technical Standards for Copy Number Variant Interpretation Concordance
Source: Front Genet. 2022 Mar 10;13:829728. doi: 10.3389/fgene.2022.829728 (PMC8960312; doi:10.3389/fgene.2022.829728)

Figure S2(A)

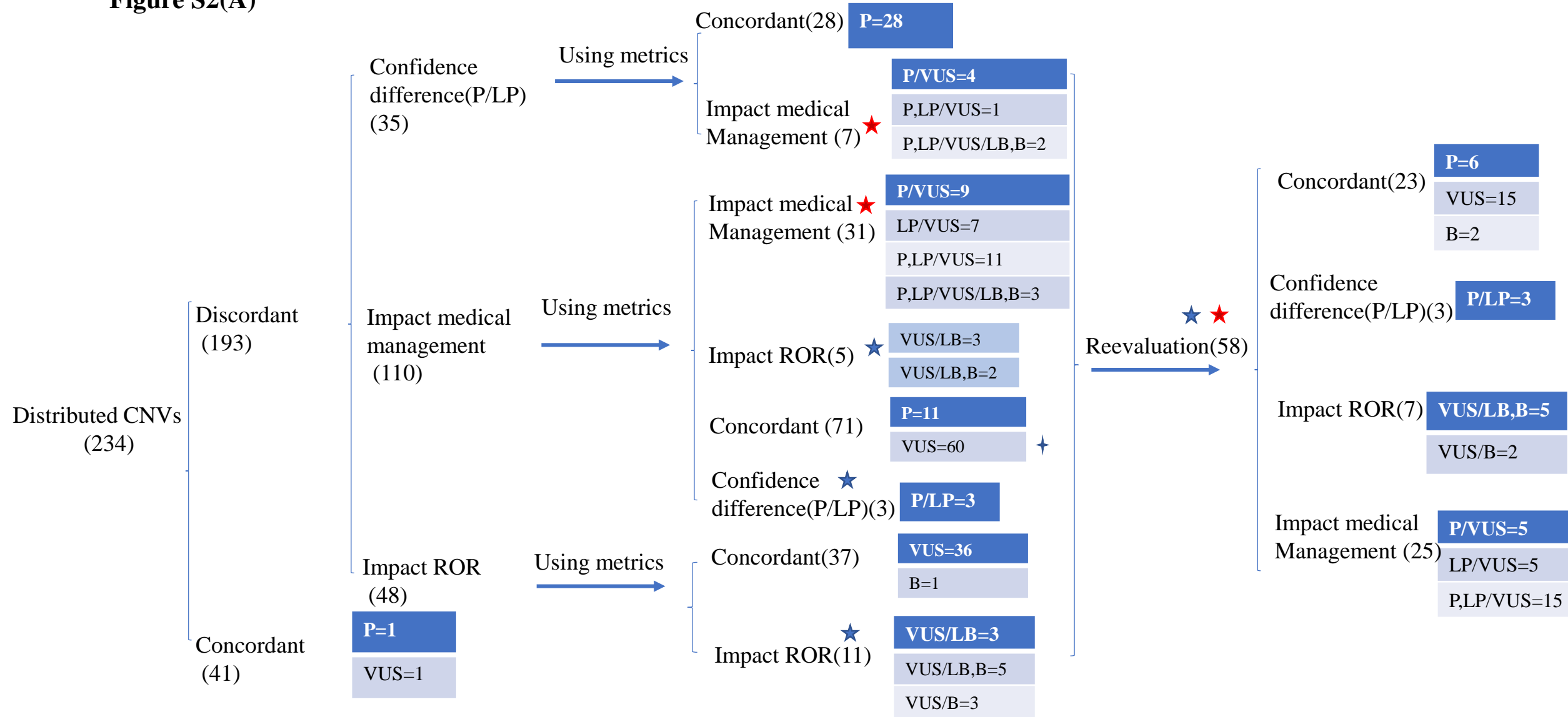

★ One concordant VUS was re-evaluated by laboratories.

Figure S2(B)

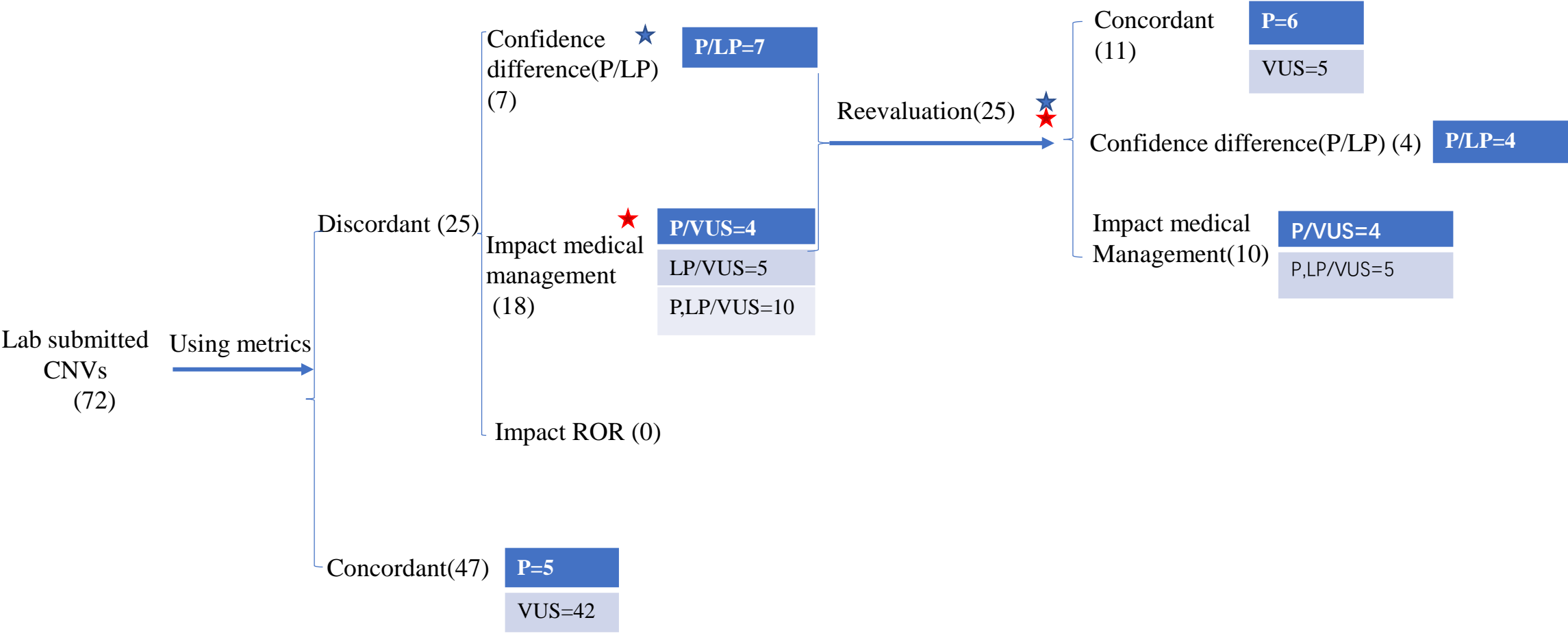

Supplement: Supplementary file 1 [file DataSheet2.PDF]

**Figure S1 A**

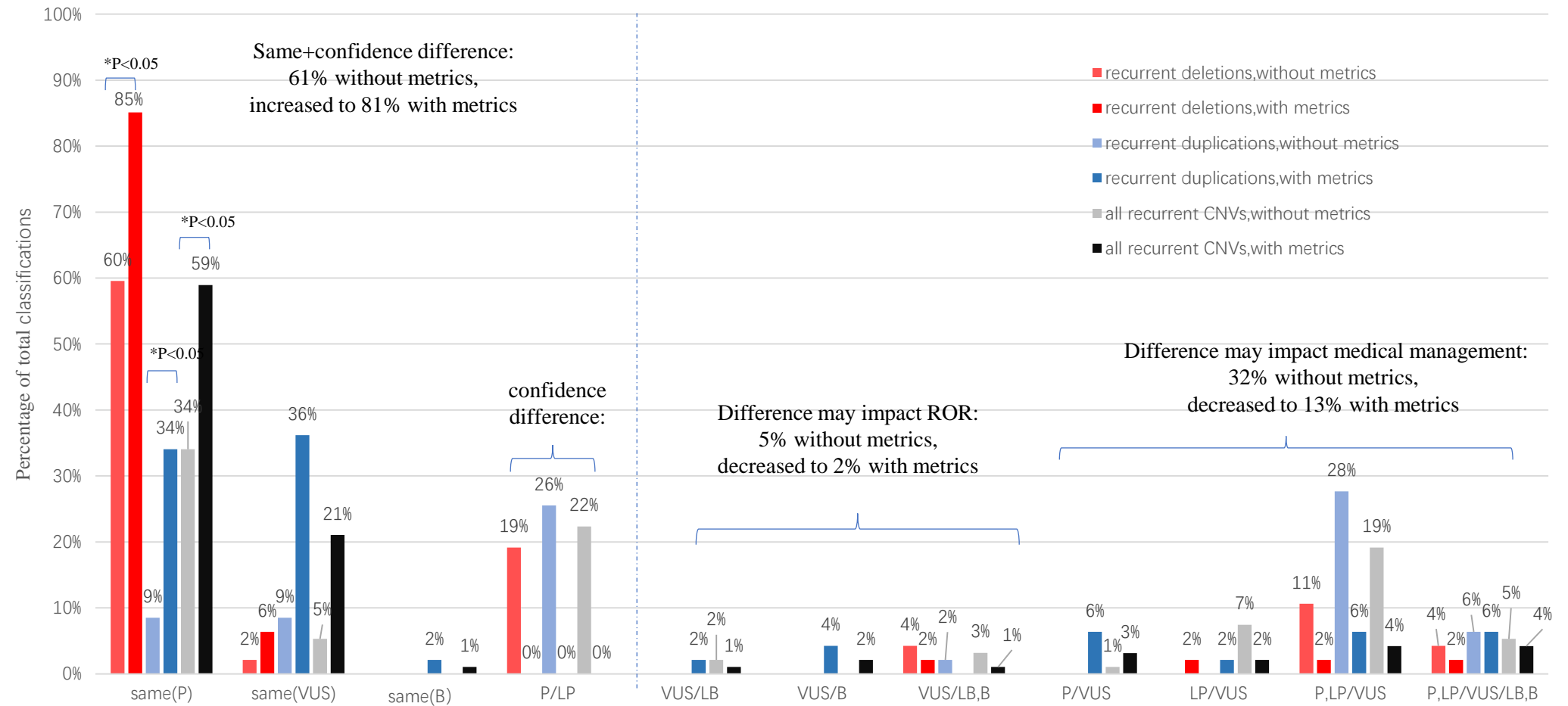

**Figure S1 B**

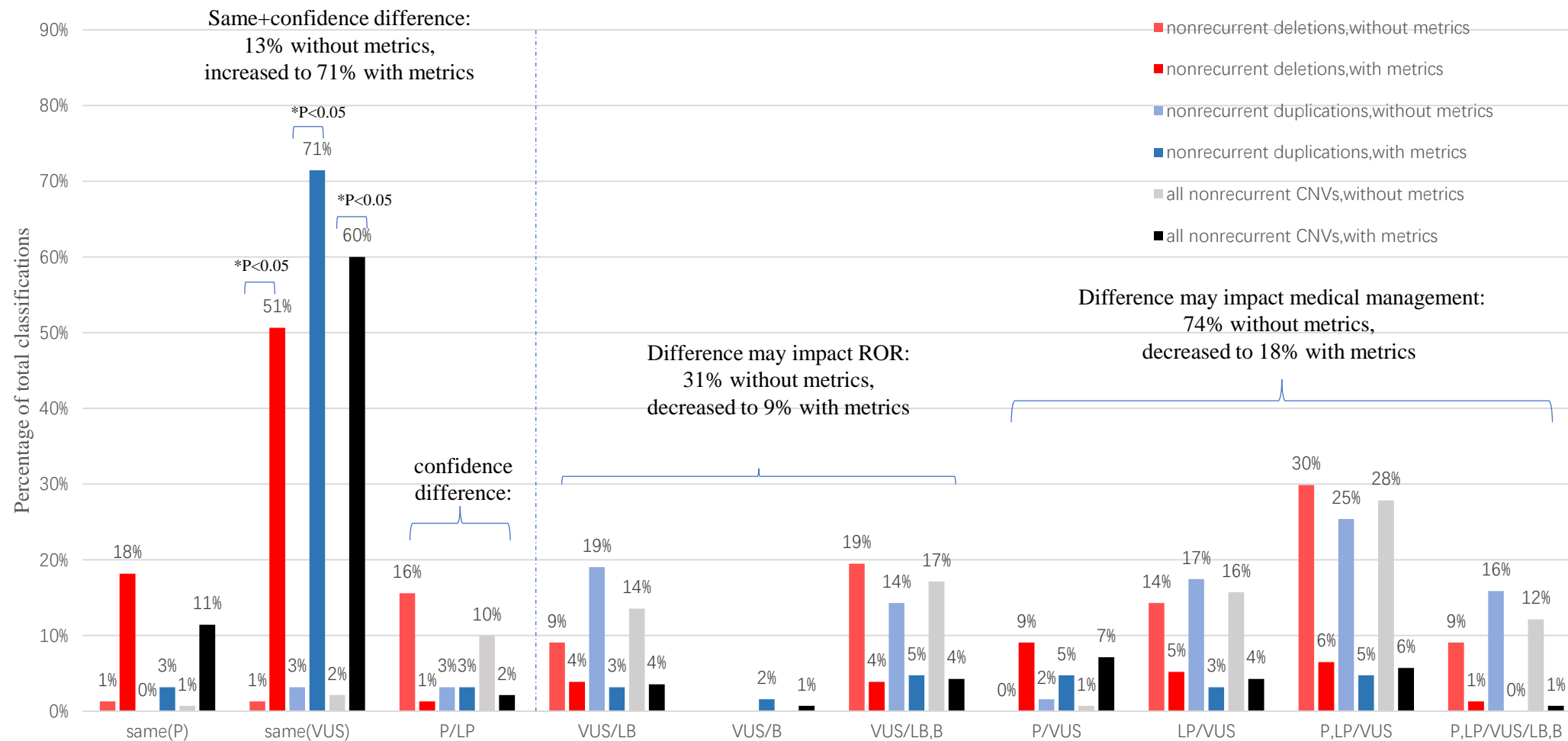

Figure S1 C

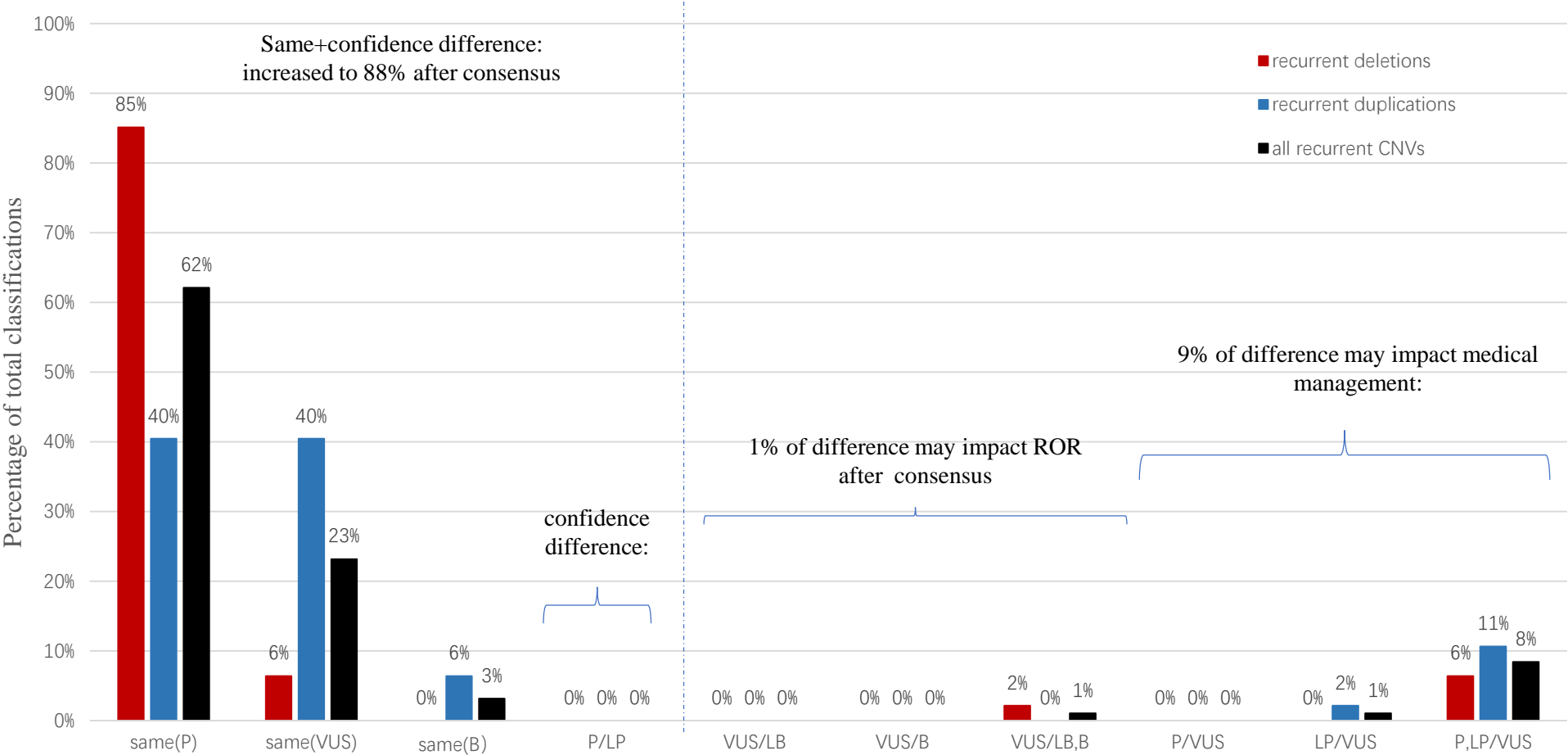

Figure S1 D

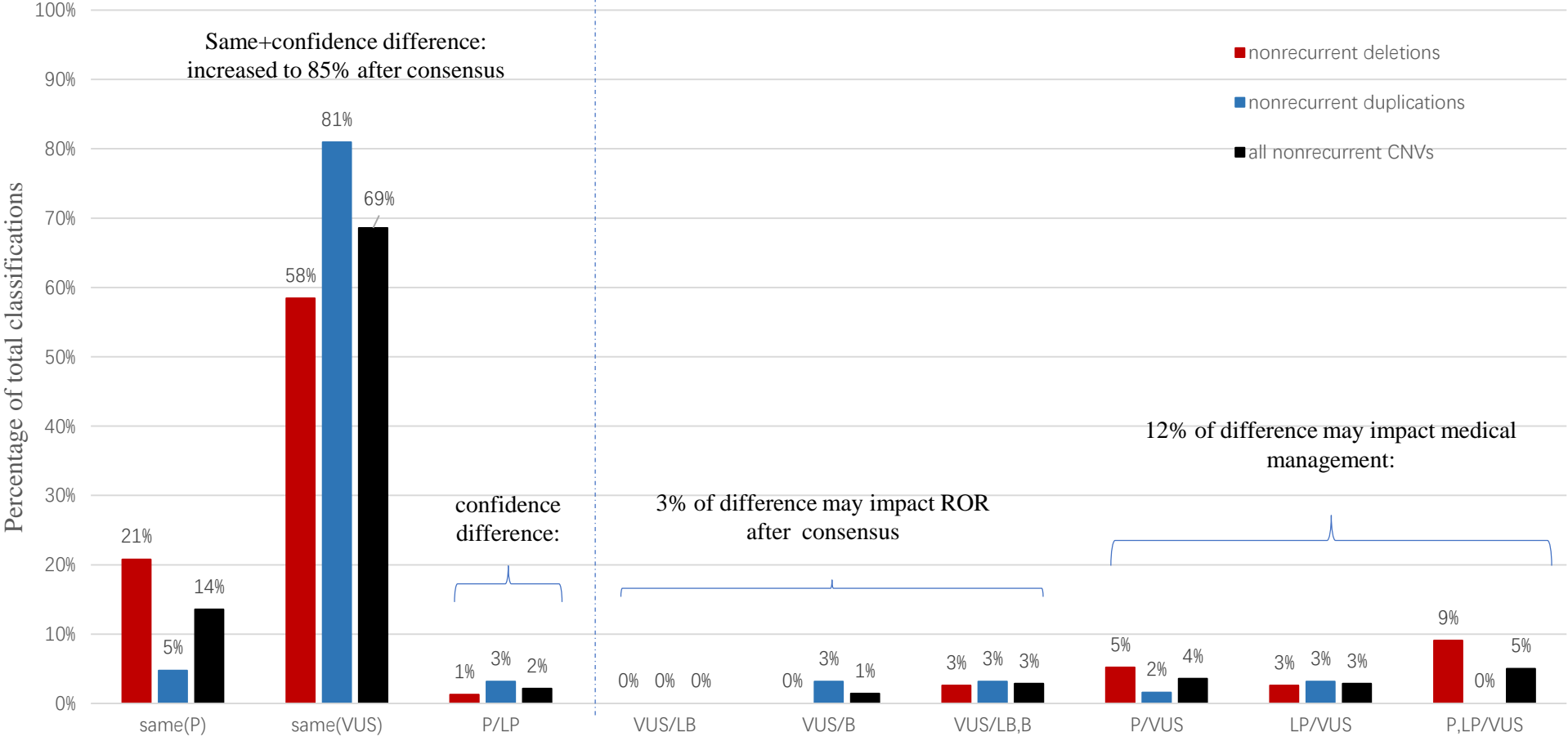

Supplement: Supplementary file 3 [file DataSheet1.PDF]
